# Brevipedicelones D and E, Two C–O–C Flavonoid Dimmers from the Leaves of *Garcinia brevipedicellata* and Anti-onchocercal Activity

**DOI:** 10.1007/s13659-018-0191-9

**Published:** 2018-12-03

**Authors:** Mirabel Akongwi, Anastasie E. Tih, Kennedy D. Nyongbela, Moses Samje, Raphael T. Ghogomu, Bernard Bodo

**Affiliations:** 1 Department of Organic Chemistry, Faculty of Science, University of Yaounde 1, P. O. Box 812, Yaounde, Cameroon; 2 Pharmacochemistry Research Laboratory, Department of Chemistry, Faculty of Science, University of Buea, P.O. Box 63, Buea, South West Region Cameroon; 3 Faculty of Science, ANDI Centre of Excellence on Onchocerciasis Research, University of Buea, P. O. Box 63, Buea, South West Region Cameroon; 4 Laboratoire de Chimie des Substances Naturelles, Muséum National d’Histoire Naturelle, 63 Rue Buffon, 75005 Paris, France

**Keywords:** *Garcinia brevipedicellata*, Brevipedicelone, Anti-onchocercal activity

## Abstract

**Abstract:**

A novel isoflavone–chromone flavonoid C–O–C dimmer, brevipedicelone D (**1**), along with one new C–O–C biflavonoid derivative, brevipedicelone E (**2**), were isolated from the ethyl acetate extract of the leaves of *Garcinia brevipedicellata*, a medicinal plant used in folk medicine in parts of Cameroon. Their structures were elucidated by extensive spectroscopic techniques, including 1D- and 2D- NMR, MS experiments, as well as comparing their spectral data with those of known analogues. Anti-onchocercal screening of **1** showed moderate inhibition of adult worm motility of *Onchocerca ochengi* by 60% at the highest concentration (20 µg/mL) and inhibited motility of both the juvenile worms of *O. ochengi* and *Loa loa* by 90% at this same concentration.

**Graphical Abstract:**

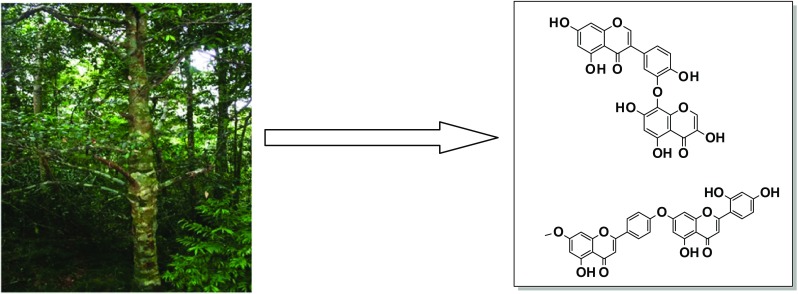

**Electronic supplementary material:**

The online version of this article (10.1007/s13659-018-0191-9) contains supplementary material, which is available to authorized users.

## Introduction

Garcinia is the most represented genus of the (Clusiaceae) family. Long known by the name Guttifreae, the Clusiaceae family is the most represented of this family, widely distributed from temperate to tropical regions and are represented by six (06) sub families which are: Kielmeyeroidae, Calophyloidae, Moronoboidae, Lorostermonoidae, Hypericoidae and Clusioidae [1]. The family at times regroups trees, shrubs, herbs and rarely lignans. Members are generally unbeared and the plants are easily recognized by their yellow or orange resinous latex which usually flows slowly when the stems, flowers and fruits are wounded while the leaves hardly produce latex [2]. Largely represented in Africa and Asia this family is made up of about 1350 species regrouped into 47 genera in low altitudes humid dense forests and are composed of six genera in Africa (i.e. *Allanblackia, Calophyllum, Garcinia, Pentadesma, Symphonia* and *Mammea*) [3]. *Garcina brevipedicellata* Oliv. is an African species that is abundant in forest regions of Cameroon and is a deciduous shrub that attains a height of about 5–9 m and about 30 cm in diameter. Previous phytochemical investigation of the leaves and stem bark of *G. brevipedicellata* have shown the presence of sterols, depsidones and tannins [4–6]. In a previous report from our lab., eight biflavonoids were isolated and characterized from the methanol extract of the stem heartwood of this plant, with three of them namely; brevipedicelones A, B, and C, being new flavonoid C–O–C dimmers [7]. This prompted further investigation of the plant.

**Fig. 1:**
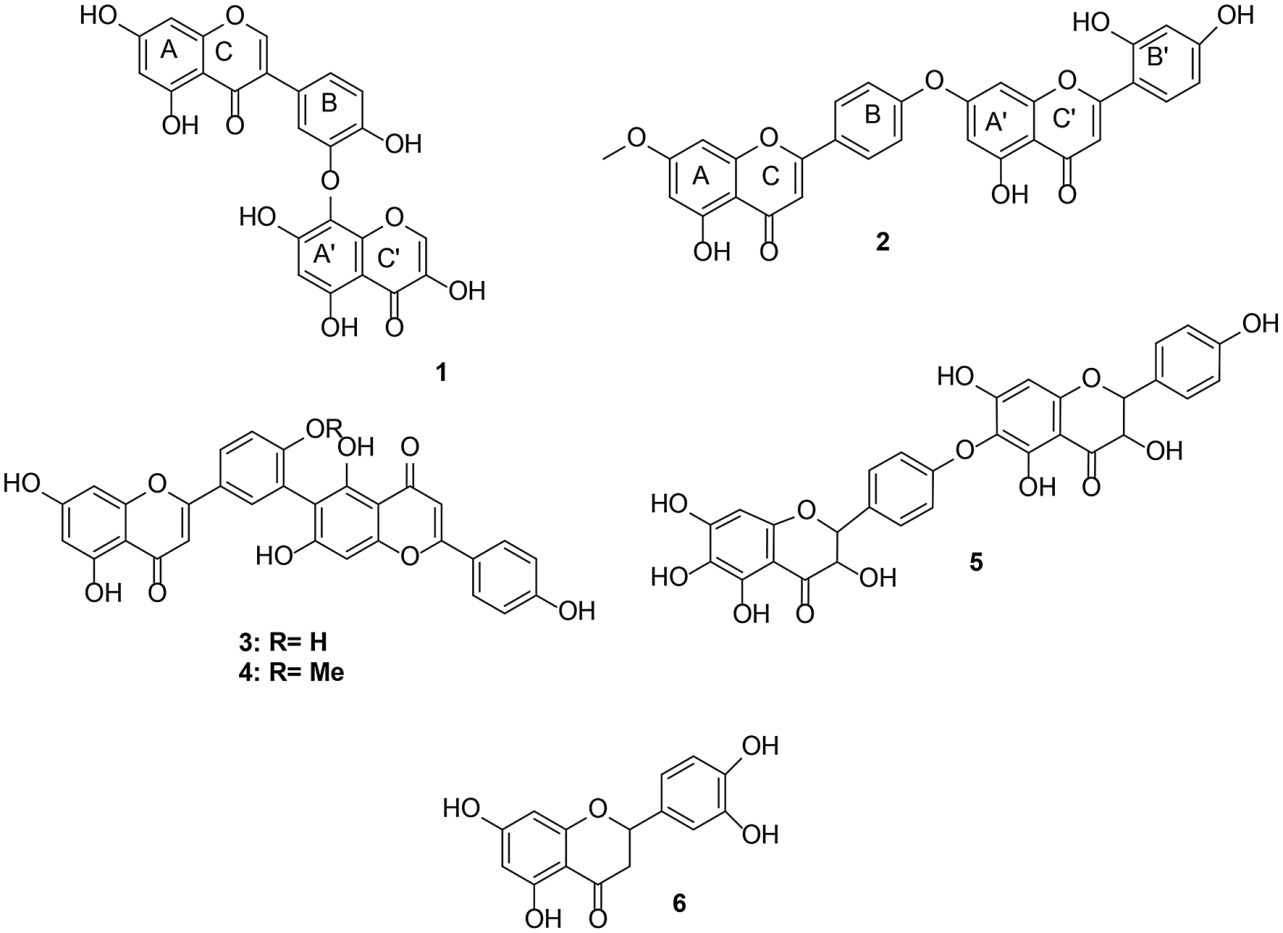
Structures of compounds **1–6**

Further investigation of the ethyl acetate extract of the leaves of this plant resulted in the isolation of a novel isoflavone–chromone flavonoid C–O–C dimmer, brevipedicelone D **(1)** and a new C–O–C biflavonoid derivative, brevipedicelone E (**2**), along with three known biflavonoids, robustaflavone (**3**), *O*-methylrobustaflavone (**4**) brevipediclone C (**5**) and one flavonoid, luteoline (**6**) (Fig. 1). We now report the isolation, structure elucidation of compounds **1** and **2** and the anti-onchocercal activity of **1** on the adult and juvenile worms (microfilariae) of *Onchocerca ochengi* and *Loa loa*, parasites responsible for human onchocerciasis (river blindness).

## Results and Discussion

The air-dried and powdered leaves of *garcinia brevipedicellata* were extracted with methanol to obtain a gum which was re-extracted with warm ethyl acetate to give a crude extract which was first fractionated by exclusion column chromatography on Sephadex LH-20. The fractions obtained were subjected to a combination of column and preparative thin layer chromatography over silica gel that led to the isolation of compounds: **1** (52 mg), **2** (10 mg), **3** (11 mg), **4** (32 mg), **5** (8 mg) and **6** (12 mg).

Compound **1** was obtained as yellow amorphous solids soluble in methanol. It gave a positive test for flavonoid (Mg/HCl). From the high resolution time of flight mass spectrum (HRTOFMS) we deduced a pseudomolecular ion peak (M+H^+^) at m/z 479.0588, corresponding to the molecular formula C_24_H_14_O_11_. The IR absorption bands indicated the existence of conjugated and chelated carbonyls (1638 cm^−1^), hydroxyl groups (3424 cm^−1^) and aromatic rings (1600 and 1498 cm^−1^). The ^1^H NMR spectrum showed a singlet at *δ*_H_ 8.37 (1H, s, H-2) indicating the presence of an isoflavone proton [8]. On the COSY spectrum, aromatic protons on ring A at *δ*_H_ 6.27 (1H, d, 2.1 Hz, H-6) and *δ*_H_ 6.46 (1H, d, 2.1 Hz, H-8) displayed *meta* coupling and were assigned to carbons C-6 and C-8 respectively. Three aromatic protons observed at *δ*_H_ 7.50 (1H, d, 2.22 Hz, H-2′), 7.13 (1H, dd, 8.46 Hz and 2.22 Hz, H-6′) and 6.65 (1H, brd, 8.46, H-5′) were attributed to carbons C-2′, C-6′ and C-5′ respectively of a trisubstituted benzynic ring (ring B). A singlet observed at *δ*_H_ 6.35 (1H, s, H-6″) was attributed to the proton on carbon C-6″ of a penta-substituted benzynic ring (ring A′).

**Fig. 2:**
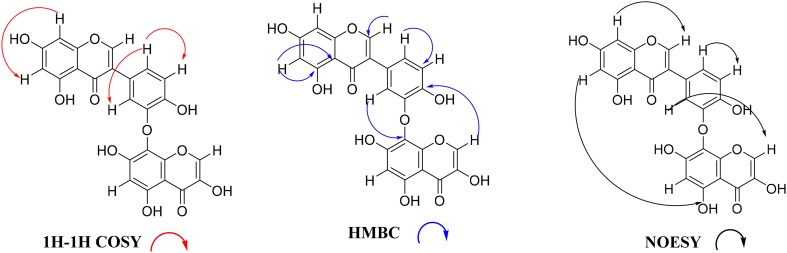
Selected COSY, HMBC and NOESY correlations of Compound **1**

In addition, a highly deshielded proton observed at *δ*_H_ 9.10 (1H, s, H-2″) suggested a chromone building block. The DEPT spectrum of **1** displayed signals for six hydroxylated carbons, six aromatic carbons and two ethylenic carbons bearing an oxygen bridge to the pyran ring of the benzopyran moieties. We also noticed in its HMBC spectrum a correlation between H-2′ and C-8″ and the axis was assigned to be located at C-3′/C8″ by a typical downfield shift of C-8″ (119.0 ppm), along with an upfield shift at C-2′ (114.5 ppm) which further confirmed the ether linkage between the two derivatives via C-3′ and C-8″ (Table 1). This ether linkage was confirmed by selected HMBC, COSY and NOESY interactions (Fig. 2). From the above spectroscopic data leading to the structure shown for compound **1**, it is characterized as a novel isoflavone–chromone flavonoid C–O–C dimmer described for the first time and named brevipedicelone D.

**Table 1:** ^1^H and ^13^C-NMR data of Compounds **1** and **2** in (*δ* in ppm, DMSO)

Compound **1**				Compound **2**			
Position	*δ*_C_	Type of carbon	*δ*_H_, mult., J(Hz)	Position	*δ*_C_	Type of carbon	*δ*_H_, mult., J(Hz)
2	154	CH	8.37 (1H,s)	2	165.0	C	–
3	122	C	–	3	101.5	CH	6.82 (1H, s)
4	180.7	C	–	4	182.0	C	–
5	161.7	C	–	5	160.5	C	12.12 (1H, s, OH)
6	99.1	CH	6.27 (1H,s, 2.10)	6	94.3	CH	6.35 (1H, d, 2.8)
7	164.4	C	–	7	167.3	C	–
8	93.9	CH	6.46 (1H,s, 2.10)	8	92.6	CH	6.77 (1H, d, 2.8)
9	157.8	C	–	9	156.3	C	–
10	104.5	C	–	10	104.6	C	–
1′	122	C	–	1′	127.6	C	–
2′	115.3	CH	7.50 (1H,d, 2.22)	2′/6′	128.1	CH	7.57 (2H, d, 8.8)
3′	146.6	C	–	3′/5′	115.6	CH	6.71 (2H, d, 8.8)
4′	147.7	C	–	4′	154.4	C	–
5′	115.4	CH	6.65 (1H,brd, 8.46)	2″	160.5	C	6.81 (1H, d, 2.2)
6′	119	CH	7.13 (1H, dd, 2.22 & 8.46)	3″	103.5	CH	6.81 (1H, d, 2.2)
2″	144.9	CH	9.12 (1H, s)	4″	181.9	C	–
3′’	135.7	C	–	5″	164.2	C	12.99 (1H, s, OH)
4″	178.2	C	–	2′′′	131.4	CH	8.04 (1H, d, 8.6)
5″	160.9	C	–	3′′′	121.4	CH	7.09 (1H, d, 8.6)
6″	98	CH	6.35(1H, s)	5′′′	102.8	CH	6.89 (1H, d, 2.8)
7″	162	C	–	OCH3-7	56.0	CH_3_	3.85 (3H, s)
8″	120.1	C	–				
9″	160.4	C	–				
10″	102.3	C	–

Compound **2** was obtained as yellow powder in CH_2_Cl_2_/MeOH (10:1) mixture. The molecular formula was C_31_H_20_O_10_ as deduced from HRTOFMS.

On its ^1^H NMR spectrum, two tetra-substituted aromatic rings with two protons exhibiting *meta* coupling signals at *δ*_H_ 6.35 (1H, d, 2.8 Hz, H-6), *δ*_H_ 6.77 (1H, d, 2.8 Hz, H-8) and *δ*_H_ 6.81 (1H, d, 2.2 Hz, H-6″ and H-8″) were attributed to rings A and A′. A para di-substituted aromatic ring with signals exhibiting an AA’BB’ spin system at *δ*_H_ 7.57 (2H, d, 8.8 Hz, H-2′ and H-6′), and *δ*_H_ 6.71 (2H, d, 8.8 Hz, H-3′ and H-5′) were attributed to ring B. Two isolated protons on a penta-substituted aromatic rings appeared at *δ*_H_ 6.82 (1H, s, H-3) and *δ*_H_ 6.89 (1H, s, H-3″) and were attributed to the flavone protons on C-3 and C-3″ of rings C and C′ respectively. A tri-substituted aromatic ring carrying three protons exhibiting an ABX spin system with signals at *δ*_H_ 8.04 (1H, d, 8.6 Hz H-2′′′), *δ*_H_ 7.09 (1H.d, 8.6 Hz, H-3′′′) and *δ*_H_ 6.89 (1H, d, 2.8 Hz, H-5′′′) was attributed to ring B′. A singlet at *δ*_H_ 3.85 that integrated for three protons was attributed to the three protons of the CH_3_O group.

On the DEPT spectrum, it was deduced that apart from the C-atom of the CH_3_O group which is saturated and whose chemical shift appears at *δ*_C_ 56.0, all the other 30 carbon atoms of the molecule are* sp*^2^ hybridized, with twelve methines (CH) [*δ*_C_ 102.4, 104.6, 94,3; 131.4, 121.4, 127.6, 102.8, 92.6, 128.1 (× 2), 115.6 (× 22)], eighteen quaternary (C) carbon atoms, ten of which carry an oxygen atom each (*δ*_C_ 154.4, 156.3 (× 2), 161.1, 160.5 (×2), 160.9, 163.5, 164.2, 165.0) and two carbonyls (*δ*_C_ 181.9 and 182.0) (Table 1).

The HMBC spectrum of **2** showed correlations between the two protons carried by ring B (H-2′ and H-6′ at *δ*_C_ 7.59) and the carbon atom at *δ*_C_ 164.5, (C-3, ring C). A first flavone sub-structure in **2** was deduced from correlations between proton H-3 (*δ*_H_ 6.82) and the C-4 (*δ*_C_ 181.9) carbonyl. Correlations were also observed on one hand between proton H-2′′′ (ring B′) at *δ*_H_ 8.04 and H-3′′ (ring C′) at *δ*_H_ 6.89 and on the other hand between the carbonyl carbon (C-4″ ring C′) at *δ*_C_ 182.0 and the proton H-3″ at *δ*_H_ 6.89 (ring C′). These led to the suggestion of a second flavone sub-structure in **2**.

On the NOESY spectrum of **2**, it was observed that there exist a correlation between H-3′ and H-5′ (*δ*_H_ 6.71, ring B) with H-6″ and H-8″ (*δ*_H_ 6.81, ring A′). This suggested that the linkage between the two flavonoid units in **2** is between C-4 (ring C) and C-7″ (ring A′). From the above spectroscopic data leading to the structure shown for compound **2**, it is characterized as a new ether biflavonoid derivative also described for the first time and named brevipedicelone E.

Biflavonoids, which are C–O–C dimmers, up to date still form a very small class of biflavonoids which for the moment have been characterized only in the Ochnaceae, Cycadaceae, Caprifoliaceae, Fabaceae and Lauraceae families. Representatives include three compounds reported from the Ochnaceae, i.e. ochnaflavone and 7″-methylochnaflavone [9], *lophirone* L [10], and *lophirone* O [11], four from the Cycadaceae, i.e. hinokiflavone [12], 7,7″-di-*O*-methyltetrahydrohinokiflavone [13], 2″,3″ dihydrohinokiflavone [14], and 2,3,2″,3″-tetrahydrohinokiflavone [15], two from the Caprifoliaceae, ioniflavone and 3′-methyl ioniflavone [16], two from the Fabaceae, beilschmieflavonoids A and B [17], and one in the Lauraceae, tepicanol A [18].

Even though the genus *Garcinia* is known as a major source of xanthones [19–26], and C–C linked flavonoid dimers [27–31], this is the first report on the characterization of a novel isoflavone–chromone flavonoid C–O–C dimmer, brevipedicelone D **(1)**, along with one new C–O–C biflavonoid derivative, brevipedicelone E **(2)**, in this genus and anti-onchocercal evaluation of compound **1** on the adult worms and microfilariae of *Onchocerca ochengi* and *Loa loa*. These characterized compounds are the first examples of flavonoid dimmers with constituent isoflavone–chromone or flavone–flavone sub-units in their structures, illustrating that the Clusiaceae family is rapidly emerging as a potential source of dimeric C–O–C flavonoids.

Compound **1** was screened on both adult and microfilaria worms of *O. Ochengi* and microfilariae (mfs) of *L. loa*. All cultures lasted for 120 h post addition of the compound. On the adult worms, the compound showed moderate activity at the highest concentration of 20 µg/ml. The compound inhibited *O. ochengi* microfilariae motility by 90% at the 20 µg/ml thus demonstrating activity at this juvenile form of the parasite. When screened on *L. loa* mfs, there was no activity at the highest concentration (Table 2). There was a dose-dependent response for the *O. ochengi* parasite that succumbed to the compound.

**Table 2:** Anti-Onchocercal activity of Compound **1** on *O. ochengi* and *L. loa*

Concentration (µg/ml)	% Inhibition of formazan formation by *O. ochengi* adult worm	% Inhibition of *O. ochengi* microfilariae motility	% Inhibition of *L. loa* microfilariae motility
20	60	90	0
10	20	50	0
5	0	25	0
2.5	0	10	0
1.25	0	0	0
0.625	0	0	0

## Experimental

### General

The mass spectra (HRTOFMS) were measured in a time of flight mode spectrometers. The ^1^H NMR spectra were registered on a 600 MHz NMR spectrometer with tetramethylsilane (TMS) as an internal standard; while ^13^C NMR spectra were recorded on a 150 MHz NMR spectrometer using DMSO as solvent. Methyl, methylene and methine carbons were distinguished by DEPT experiments. Homonuclear ^1^H connectivities were determined by using the COSY experiment. One-bond ^1^H–^13^C connectivities were determined with HMQC gradient pulse factor selection. Two and three bonds ^1^H-^13^C connectivities were determined by HMBC experiment. Chemical shifts were reported in *δ* (ppm) and coupling constants (J) were measured in Hz.

### Plant material

The leaves of *Garcinia brevipedicellata* were harvested in August 2013 in Malande a village situated at 3 km from DIBANG sub-division in the Nyong et Kelle Division of the Centre Region, Cameroon and identified by Mr. Victor Nana, botanist of the Cameroon National Herbarium Yaounde, Cameroon where a voucher specimen (No.VN 2634) was deposited.

### Extraction and Isolation

Air dried and ground leaves of *Garcinia brevipedicellata* (1.5 kg) were extracted using methanol. After evaporating the solvent the methanolic residue obtained was exhaustively fractionated into three, by extraction with three solvents in increasing polarity. First with ethyl acetate, followed with acetone and lastly with methanol. Only the ethyl acetate fraction (62 g) was investigated. This extract was divided into four equal portions A-D and each portion fractionated by size exclusion chromatography on Sephadex gel LH-20 and eluted with methanol. Sub-fractions were pooled together to obtain five main fractions. N_1_ (6.1 g), N_2_ (4.3 g), N_3_ (34 g), N_4_ (5.5 g) and N_5_ (3.2 g). Fraction N_4_ was purified twice by chromatography on a silica gel column with CH_2_Cl_2_/MeOH (10:1) to afford Compound **1** (52 mg). N_3_ and N_2_ were repeatedly purified on an open silica gel column with CH_2_Cl_2_/MeOH (10:1) followed by preparative thin layer chromatography to give Compounds **2** (10 mg), **3** (11 mg) and **4** (32 mg), **5** (8 mg), **6** (12 mg) and more of compound **3** (8 mg) respectively.

#### Brevipedicelone D (1)

Yellow amorphous powder, UV (MeOH): λmax (log ε) = 268 and 316 nm, IR (KBr): ν_max_:1638, 3424, 1600 and 1498 cm^−1^ ^1^H and ^13^C NMR spectroscopic data, see Table 1; HRTOFMS (pos.) m/z 479.0588, (M+H^+^) (calc. for C_24_H_15_O_11_, 478.0536).

#### Brevipedicelone E (2)

Yellow powder, UV (MeOH): λmax (log ε) λmax 223 and 320 nm, IR (KBr): ν_max_: 3234, 1638 1628 cm^−1^), 1603 and 1508 cm^−1^. ^1^H and ^13^C NMR spectroscopic data, see Table 1; HRTOFMS (pos.) m/z, 553.0960 (M+H^+^) (calc. for C_31_H_21_O_10_, 552.1122).

### Anti-onchocercal Screening

#### Extraction of *Onchocerca ochengi* Adult Worms

*Onchocerca ochengi* adult worm masses were extracted from cattle skin by the method employed in [32]. Briefly, fresh pieces of umbilical cattle skin containing palpable nodules were obtained from local slaughterhouse in Buea, Cameroon. The piece of skin was immediately transported to laboratory. The skin was thoroughly washed with soap and distilled water, drained, dried by blotting with a piece of cloth and then transferred to a sterile laminar flow hood. It was then entirely covered with 70% ethanol and allowed to evaporate completely on its own. The nodules were carefully dissected using a sterile razor blade and the pale orange-yellow worms (in appearance) were immediately submerged in sterile 12-well culture plates (NUNC, USA) containing 2 ml of complete culture medium [RPMI-1640 supplemented with 25 mM HEPES, 2 g/L sodium bicarbonate, 20 mM l-glutamine, 10% new born calf serum (SIGMA, USA), 2 × antibiotic–antimycotic (Sigma, USA)], pH 7.4]. After overnight culture, 1 mL of medium was added before addition of drug making a total volume of 3 mL. Adult worm cultures were carried out at 37 °C under an atmosphere of 5% CO_2_ in humidified air in an incubator (HERACell 150, Haraeus, Germany).

#### Extraction of *Onchocerca ochengi* Microfilariae

The microfilariae of *O. ochengi* were extracted by the method of [33], with some slight modifications. Cattle skin got from the slaughterhouse was thoroughly cleaned and sterilized as above. The skin was then firmly attached onto a sterilized flat wooden board using autoclaved thumbnails. The outer surface was carefully shaved with a sterile razor blade, and then rinsed twice with distilled water. A clean dry adsorbent cloth was used to remove excess moisture from the skin. The entire skin was covered with 70% ethanol and allowed to evaporate in a laminar flow hood. This sterilization process was done twice. Once the alcohol had completely evaporated from the skin, skin snips were obtained from different locations of the skin. These sleeves were carefully scrapped and the snips submerged in 15 mL of complete culture medium. The assemblage was incubated at room temperature for 2 h to allow for emergence of microfilariae. The highly motile microfilariae that emerged were concentrated by centrifugation at 400×*g* for 10 min. The supernatant was decanted, and the pelleted mfs were re-suspended in fresh complete culture medium. The highly motile microfilariae were quantified using an inverted microscope (Euromex, Holland). One hundred microlitres of culture medium containing microfilariae were distributed into 96 well culture plate containing LLC-MK2 cell layers to obtain an average of 12–15 mfs per well. Culture conditions were the same as that of adult worms above.

#### Isolation and Culture of *Loa loa* Microfilariae

Blood was collected from *Loa loa* infected individuals at the Edea Health District after confirmation by Giemsa stain. The blood was rapidly transferred to the University laboratory. The microfilariae were isolated by the method of [32]. Freshly collected *L. loa* -infected blood was diluted (1:2) with culture media used above but without sera. The diluted blood was carefully layered on 4 mL of Ficoll-pacque (TM) in a 15 mL centrifuge tube. The tube was spun in a swing bucket centrifuge at 400×*g* for 15 min. The recovered mfs were washed three times with culture media (without sera) and then re-suspended in media containing sera. The mfs were then distributed in wells of a 96-well microtiter plate containing LLC-MK2 cell layers. Each well contained 12–15 mfs in 100 of media.

#### Preparation of Compound and Assessment of Activity

Compound **1** was dissolved in ≥ 99.9% sterile dimethyl sulfoxide (SIGMA, USA) giving a stock concentration of 5 µg/ml. The compound was prepared at 2× the final concentration and distributed to wells containing parasites. For the microfilariae, 100 µL was added while 1 mL was added to wells containing adult worms to give a final volume of 200 µL and 4 mL for microfilariae and adult worms respectively. All the cultures were conducted for 120 h post addition of the compound. Auranofin [34], served as positive control for adult worm assay while ivermectin and amocarzine were used as positive control drugs for *O. ochengi* and *L. loa* mfs respectively. The diluent (dimethyl sulfoxide) was added to the negative control wells. Inhibition of microfilariae motility was assessed using an inverted microscope. Effect of compound on adult worm viability was assessed using the MTT-formazan assay following procedures employed by [32, 33].

#### Ethical Issues

We got ethical clearance (2013/11/371/L/CNERSH/SP) and administrative authorization (631–06.14) from respectively, the Cameroon National Ethical Committee and the Ministry of Public Health, Cameroon. Local administrative authorization was also obtained from the District Medical Officer of the Edea Health District. Informed consent was obtained freely from individuals who harbored high *L. loa* mf load.

## Electronic supplementary material

Below is the link to the electronic supplementary material.

Supplementary material 1 (DOCX 2511 kb)
