# Supplementary material for: Brevipedicelones D and E, Two C–O–C Flavonoid Dimmers from the Leaves of Garcinia brevipedicellata and Anti-onchocercal Activity
Source: Nat Prod Bioprospect. 2018 Dec 3;9(1):61–8. doi: 10.1007/s13659-018-0191-9 (PMC6328427; doi:10.1007/s13659-018-0191-9)
Supplement: Supplementary file 1 — Supplementary material 1 (DOCX 2511 kb) [file 13659_2018_191_MOESM1_ESM.docx]

Supplementary data for Compound **1**

**Mass Spectrum**


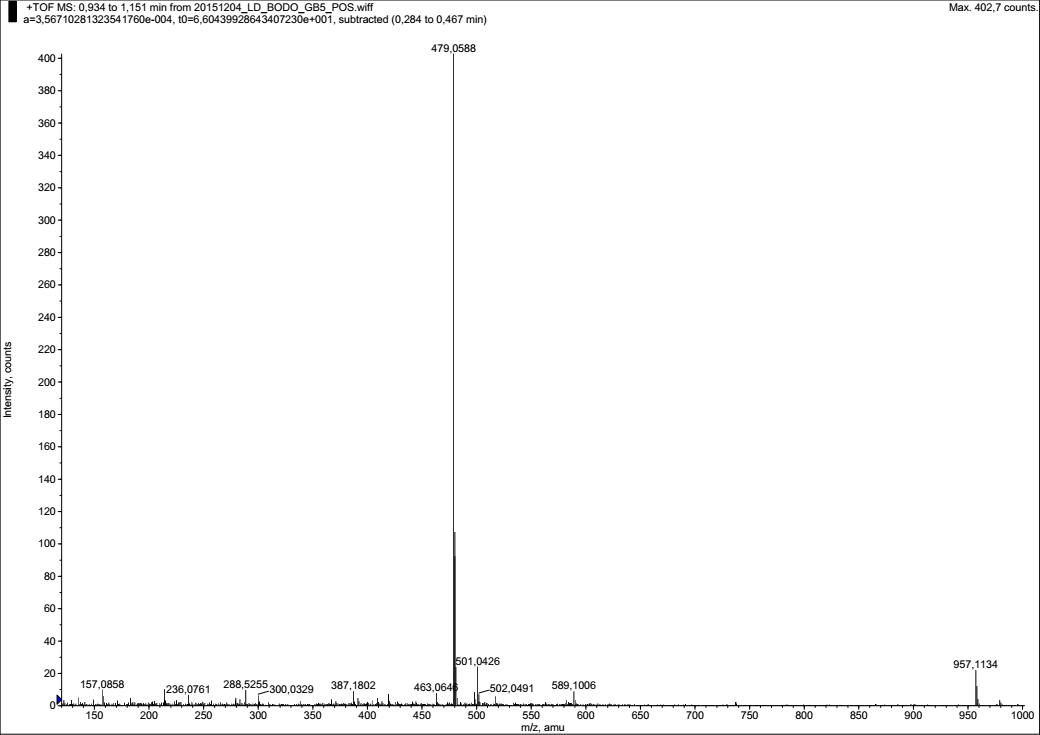


**^1^H NMR (DMSO/298K)**


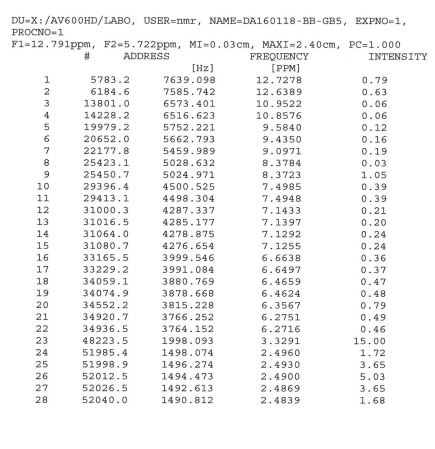


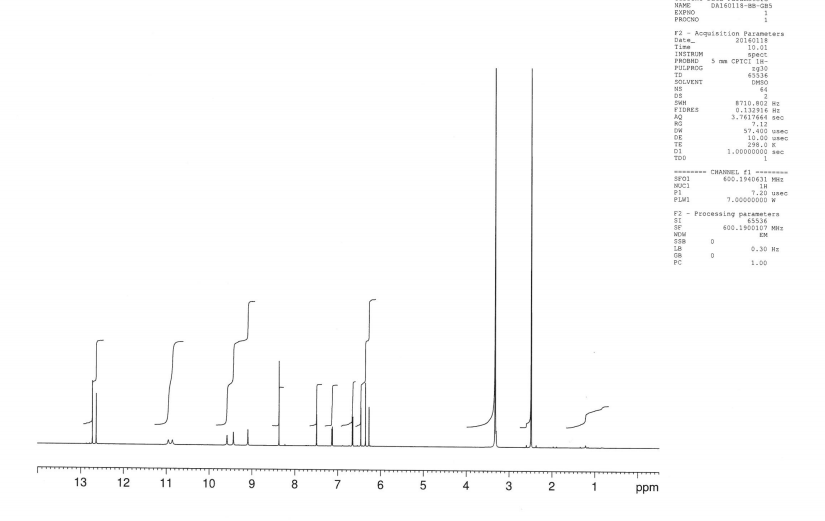


**COSY/ DMSO/298K**


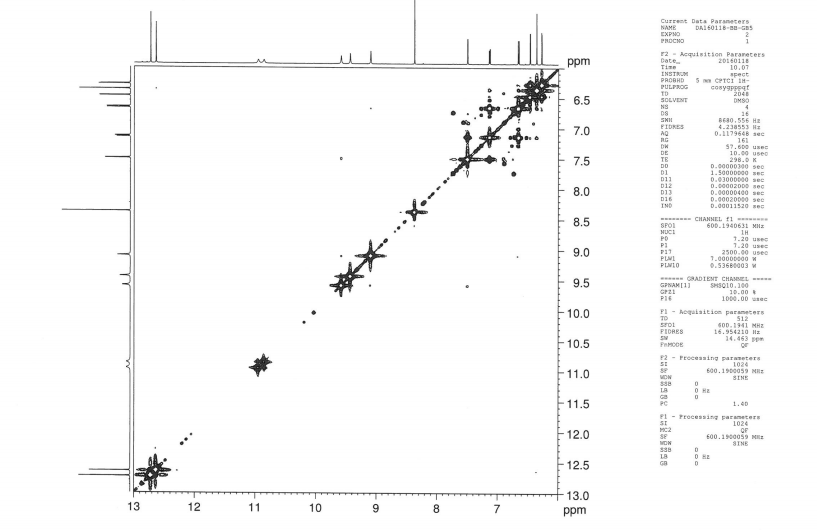


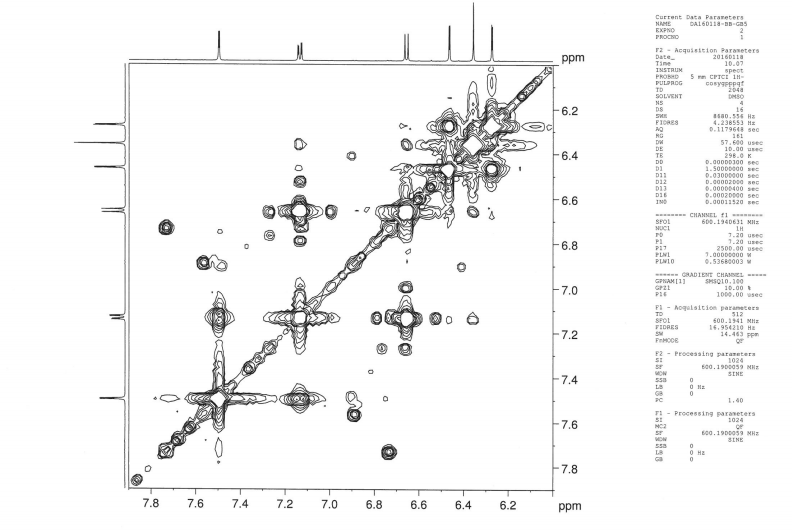


**HMBC/ DMSO/298K**


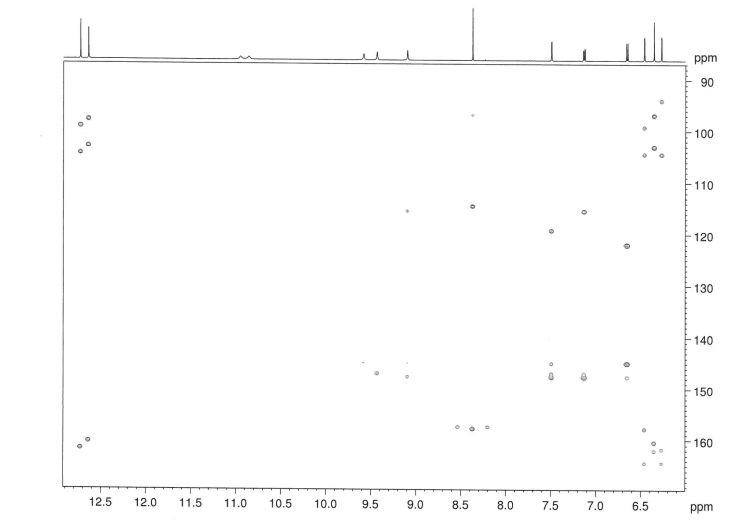


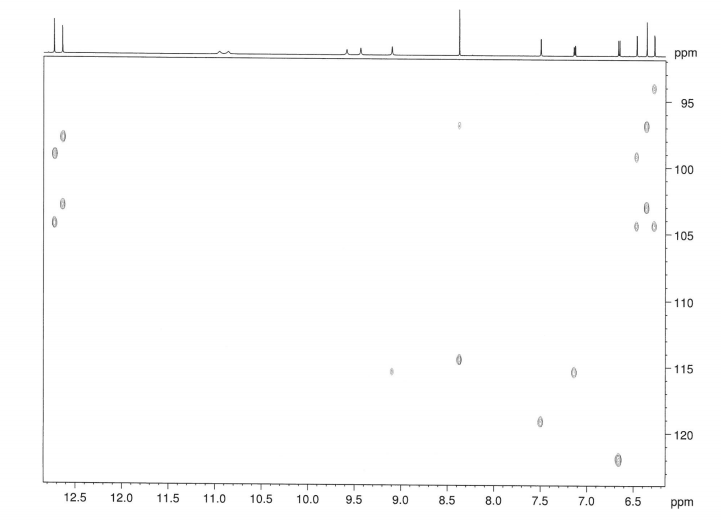


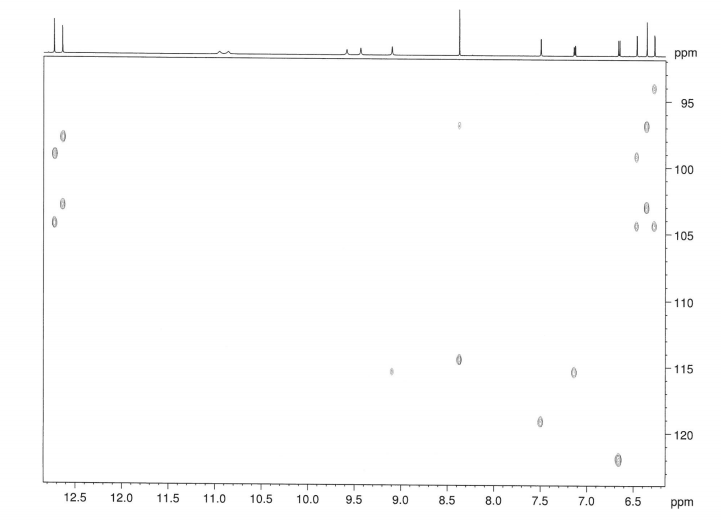


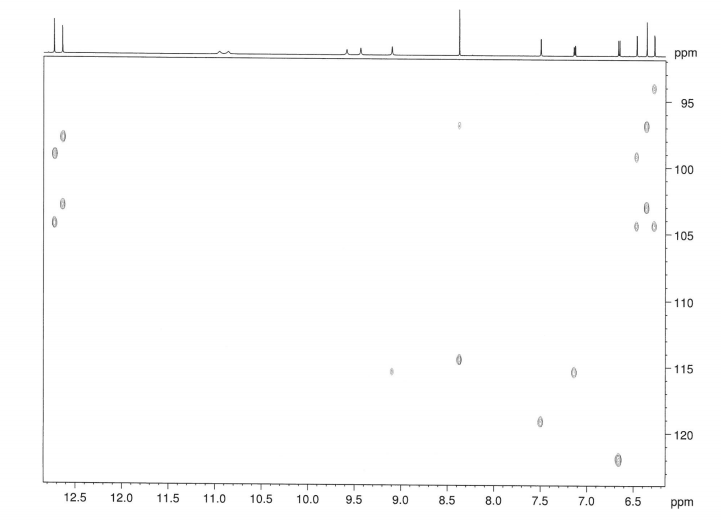


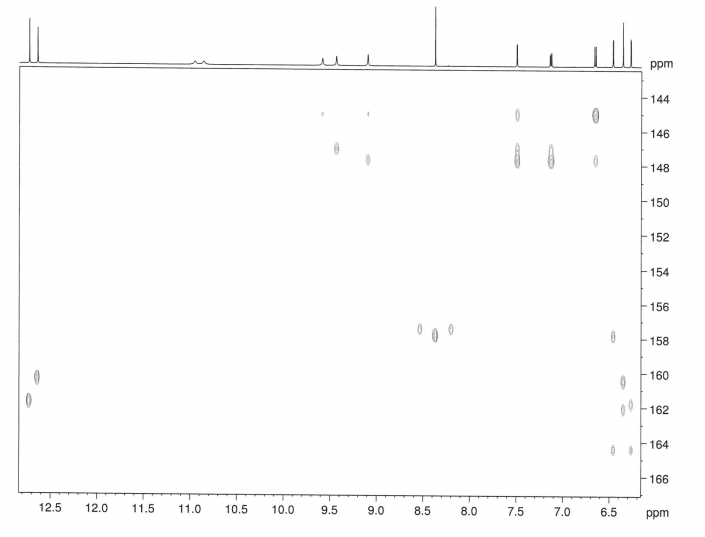

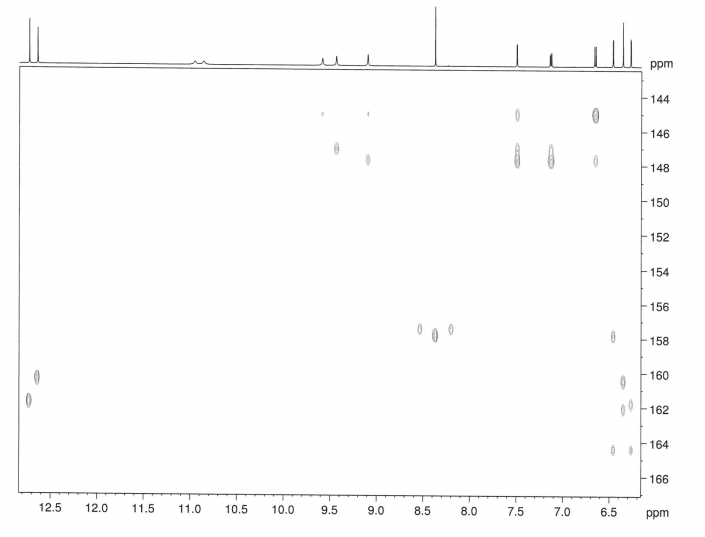


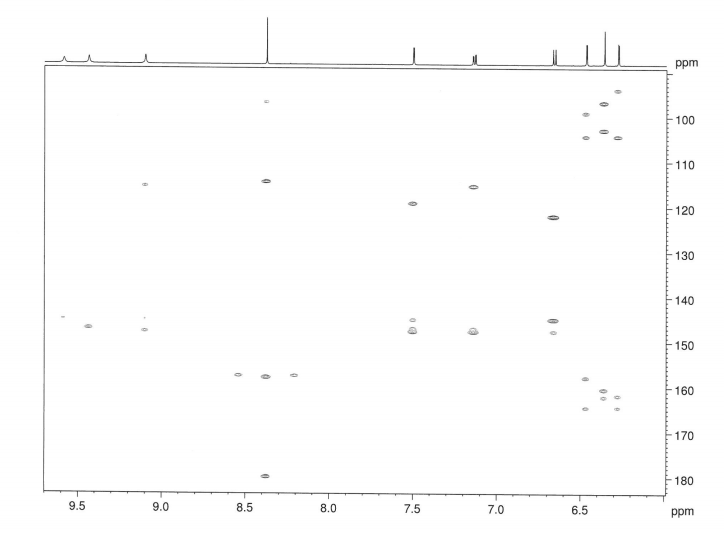


HSQC/ DMSO/298K


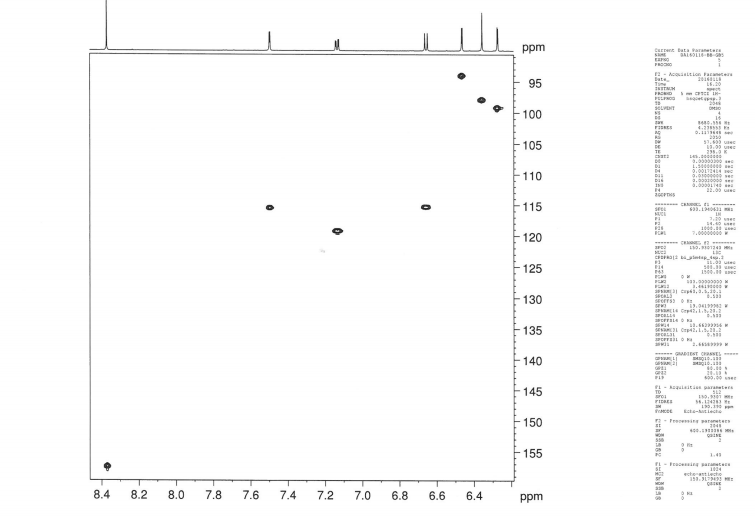


NOESY/ DMSO/298K


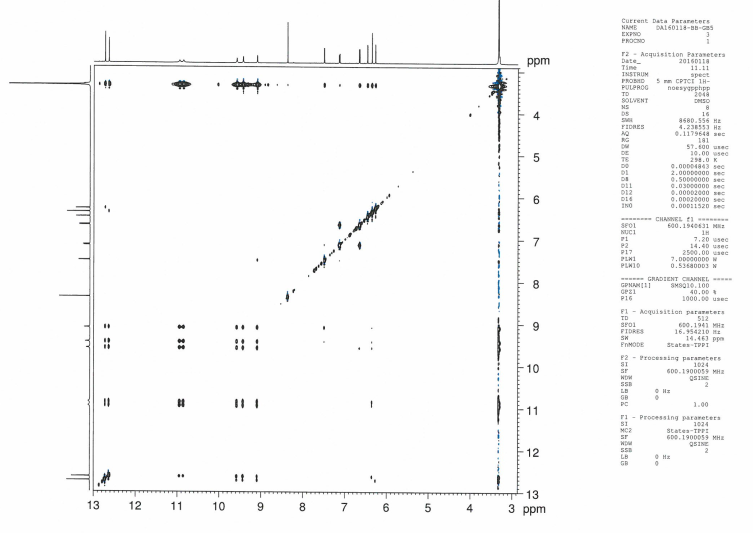


DEPT Q/ DMSO/298K


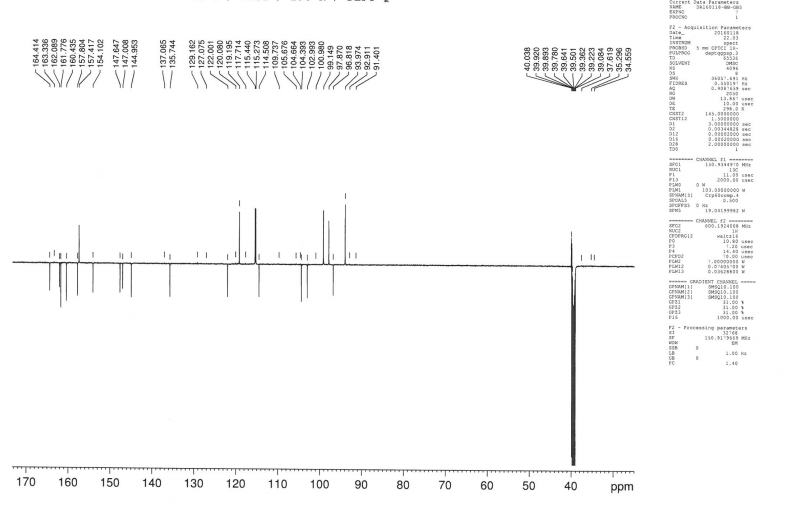


Supplementary data for Compound **2**

**Mass Spectrum**

**
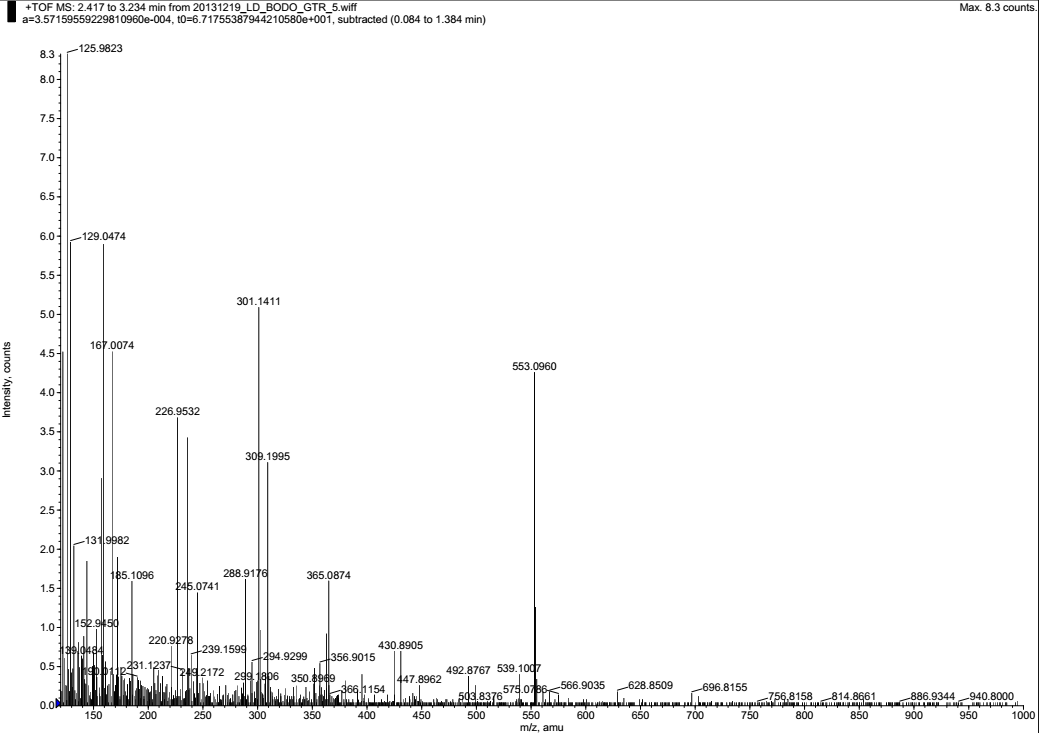
**

**^1^H NMR**


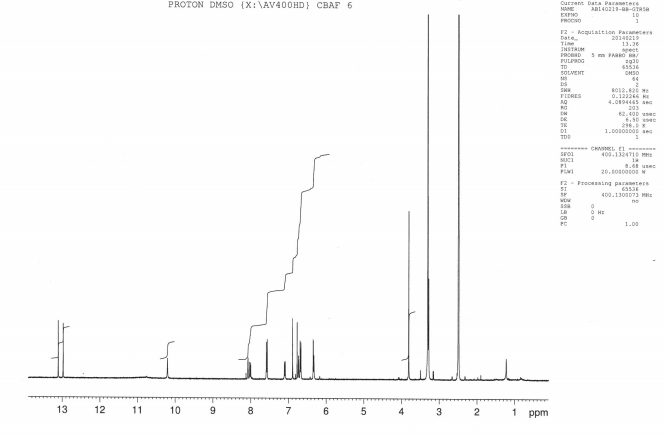


**COSY/DMSO/298K**

**
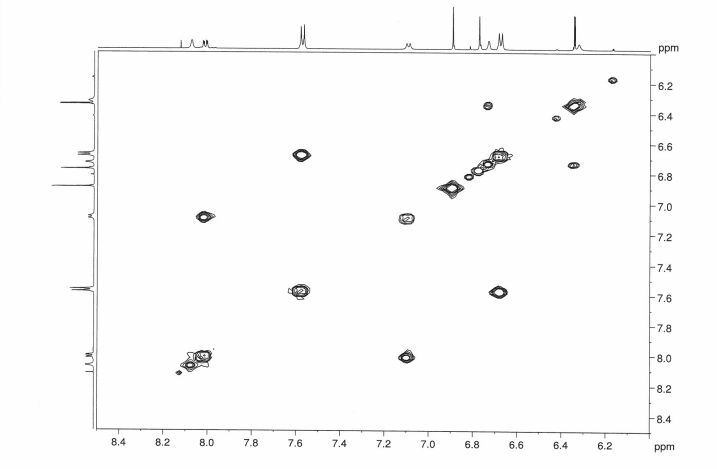
**

**HMBC/DMSO/298K**

**
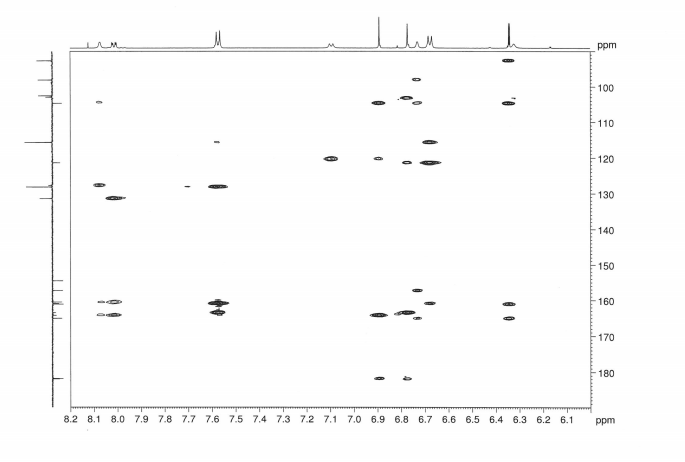
**

**DEPT Q/DMSO/298K**

**
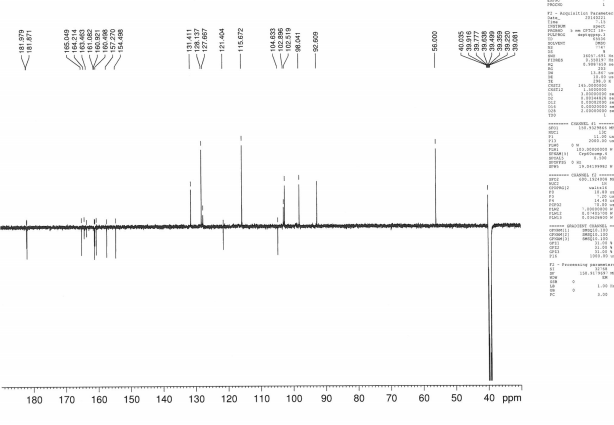
**

**HSQC/DMSO/298K**

**
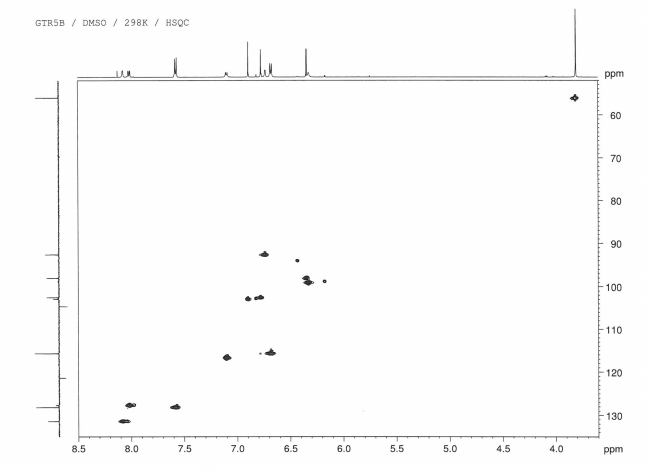
**

**NOESY/DMSO/298K**
